# The Role of Vitamin D_3_ in Periodontal Health: Implications for Bone Metabolism, Immune Modulation and Inflammation Control

**DOI:** 10.3390/nu18040577

**Published:** 2026-02-10

**Authors:** Julia Moszura, Sebastian Gawlak-Socka, Jakub Pęksa, Natalia Bielecka-Kowalska, Sebastian Kłosek

**Affiliations:** 1 Student Scientific Association at the Department of Oral Mucosal and Periodontal Diseases, Medical University of Lodz, Pomorska 251, 92-213 Lodz, Poland; julia.moszura@student.umed.lodz.pl (J.M.); sebastian.gawlak-socka@student.umed.lodz.pl (S.G.-S.); jakub.peksa@student.umed.lodz.pl (J.P.); 2 Student Scientific Association at the Department of Maxillofacial Surgery, Medical University of Lodz, Pomorska 251, 92-213 Lodz, Poland; 3 Department of Oral Mucosal and Periodontal Diseases, Medical University of Lodz, Pomorska 251, 92-213 Lodz, Poland; sebastian.klosek@umed.lodz.pl; 4 Department of Oral Pathology, Medical University of Lodz, Pomorska 251, 92-213 Lodz, Poland

**Keywords:** vitamin D_3_, periodontal disease, immune modulation, anti-inflammatory, bone metabolism, clinical outcomes

## Abstract

Vitamin D_3_ is a fat-soluble steroid essential for bone metabolism, immune modulation, and inflammation control, all critical for periodontal health. Its active form, 1,25-dihydroxyvitamin D_3_, binds to the vitamin D receptor (VDR) in periodontal cells, including periodontal ligament stromal cells, fibroblasts, osteoblasts, and macrophages, enhancing osteogenesis, antimicrobial defenses, and anti-inflammatory responses. Clinical and experimental evidence demonstrates that adequate systemic vitamin D_3_ levels and local activation in gingival tissues improve outcomes of nonsurgical and surgical periodontal therapies, reducing probing pocket depth (PPD), clinical attachment loss (CAL), and gingival inflammation. Dose-dependent supplementation shows greater clinical efficacy, and emerging evidence supports potential topical applications. This review integrates molecular mechanisms with clinical findings, highlighting the therapeutic potential of vitamin D_3_ in periodontal disease management.

## 1. Introduction

Vitamin D is a fat-soluble steroid that exists in two forms: vitamin D_2_ and vitamin D_3_ (cholecalciferol), which are synthesized from different precursors. Vitamin D_2_ is produced from ergosterol, primarily by animals and plants, while vitamin D_3_ is synthesized from 7-dehydrocholesterol (7-DHC), primarily in humans [1,2]. Vitamin D_3_ is then transported to the liver, where it is converted by the enzyme CYP2R1 into 25-hydroxyvitamin D_3_ [25(OH)D_3_], the main circulating form of vitamin D_3_ [3]. In the kidneys, this form is further converted into its active form, 1,25-dihydroxyvitamin D_3_ [1,25(OH)_2_D_3_], through the action of CYP27B1 [4].

Optimal serum concentrations of 25-hydroxyvitamin D are generally considered to be ≥30 ng/mL (≥75 nmol/L), a level widely accepted as sufficient for maintaining skeletal health and proper immune function. Serum levels ≤30 ng/mL are classified as sub-physiologic and are associated with impaired calcium–phosphate metabolism, altered immune responses, and increased susceptibility to inflammatory diseases [5]. This active form of vitamin D plays a critical role in various bodily functions. It also serves an immune function by regulating the proliferation and differentiation of cells across different cell lineages [5].

Vitamin D_3_ plays a crucial role in the prevention and treatment of periodontal diseases through various mechanisms. It is essential for the regulation of the immune response and the reduction in inflammation, which are critical factors in combating periodontal infections [6]. A deficiency in vitamin D_3_ is associated with an increased risk and severity of periodontitis, as it impairs immune function, allowing for uncontrolled bacterial growth and inflammation within the periodontal pocket [7]. The anti-inflammatory properties of vitamin D_3_ further contribute to a healing-conducive environment by modulating cytokine production [6]. Its significance also extends to bone health, as it regulates calcium levels and supports bone mineralization, which is key to preventing bone loss associated with periodontal disease [8].

**Figure 1:**
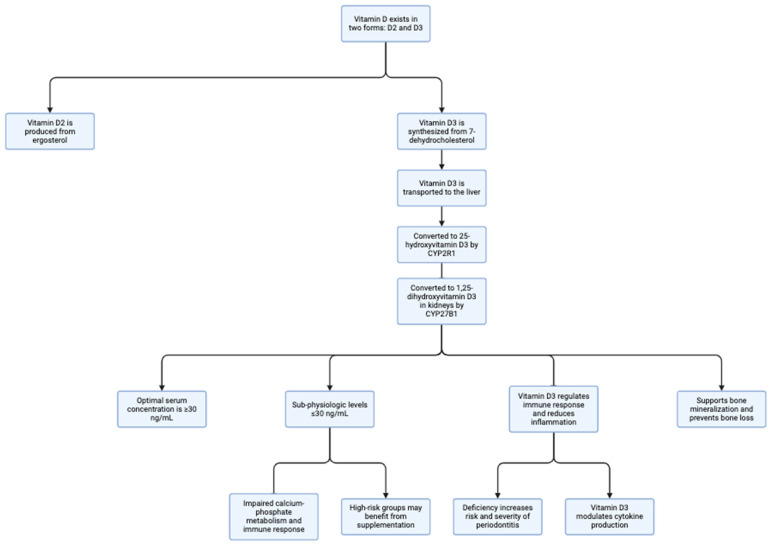
Metabolism of vitamin D and its role in immune regulation, bone homeostasis, and periodontal health. Overview of vitamin D metabolism and its biological significance in immune function, bone metabolism, and periodontal health. Vitamin D exists in two main forms: vitamin D_2_, derived from ergosterol, and vitamin D_3_, synthesized from 7-dehydrocholesterol. Vitamin D_3_ is transported to the liver, where it is converted by CYP2R1 into 25-hydroxyvitamin D_3_ [25(OH)D_3_], the principal circulating form. Subsequent renal hydroxylation by CYP27B1 generates the biologically active form, 1,25-dihydroxyvitamin D_3_ [1,25(OH)_2_D_3_]. Optimal serum concentrations of 25(OH)D_3_ (≥30 ng/mL) support proper calcium–phosphate metabolism, immune regulation, and bone mineralization. Sub-physiologic levels (≤30 ng/mL) are associated with impaired immune responses, dysregulated inflammation, and increased susceptibility to periodontal disease. Vitamin D_3_ modulates cytokine production, reduces inflammatory responses, and supports alveolar bone preservation, while individuals from high-risk groups may benefit from supplementation as an adjunct to periodontal therapy. Created in BioRender. Gawlak-Socka, S. Figure 1. 2026. Available online: https://BioRender.com/832opio (accessed on 19 January 2026) [11].

Furthermore, vitamin D_3_ is essential for bone metabolism and mineralization, processes that are directly relevant to the preservation of alveolar bone and the prevention of periodontal bone loss [9]. Individuals with suboptimal serum vitamin D levels (≤30 ng/mL), as well as those belonging to high-risk groups—such as smokers, patients with diabetes, obesity, or compromised immune function, may particularly benefit from vitamin D supplementation as an adjunct to periodontal therapy (Figure 1) [10].

This manuscript aims to provide a comprehensive analysis of the role of vitamin D_3_ in the maintenance of periodontal health, with a specific focus on three main mechanisms: bone metabolism, immune modulation, and inflammation control. We integrate recent findings and clinical trials with foundational knowledge in physiology and pathophysiology, positioning vitamin D_3_ as a potential adjunct in periodontal therapy.

Despite the existence of previous reviews addressing the role of vitamin D in periodontal disease, many focus predominantly on epidemiological associations or immune-related mechanisms. This review expands upon existing literature by integrating evidence on bone metabolism, immune modulation, and inflammation control, while incorporating recent experimental and clinical studies that highlight vitamin D_3_ as a potential adjunctive factor in both nonsurgical and surgical periodontal therapy.

### Review Strategy and Literature Section

This narrative review was conducted to synthesize current knowledge on the role of vitamin D_3_ in periodontal health, with particular emphasis on bone metabolism, immune modulation, and inflammation control. The topics covered in this review were identified through an initial exploratory literature search and iterative discussion among the authors to establish a biologically and clinically coherent framework.

A comprehensive literature search was performed using the PubMed and Google scholar. The search included articles published in English from 2001 up to 2026. Keywords included combinations of: “vitamin D_3_”, “25-hydroxyvitamin D”, “periodontal disease”, “periodontitis”, “gingivitis”, “alveolar bone”, “immune modulation”, “inflammation”, “antimicrobial peptides”, and “periodontal therapy”. Additional relevant studies were identified by screening reference lists of key publications.

Both experimental (in vitro and animal) and clinical human studies were included to provide a comprehensive overview of the biological mechanisms and clinical relevance of vitamin D_3_ in periodontal tissues. This integrative approach was adopted to bridge mechanistic insights with clinical observations, particularly in areas where randomized clinical trials remain limited. Studies focusing on skeletal bone metabolism and osteoporosis were also included when they provided relevant mechanistic or clinical parallels to alveolar bone remodeling.

As this was a narrative review, no formal inclusion or exclusion criteria or quantitative synthesis were applied. Instead, studies were selected based on relevance, methodological quality, and contribution to understanding the multifaceted role of vitamin D_3_ in periodontal health.

## 2. Vitamin D_3_ and Bone Metabolism in the Periodontium

Vitamin D_3_ plays a crucial role in the regulation of bone metabolism, which directly influences periodontal health. Upon conversion to its active form (1,25-dihydroxyvitamin D, calcitriol), it facilitates the absorption of calcium and phosphorus from the gastrointestinal tract, thereby ensuring the maintenance of adequate serum levels of these essential minerals [7,8]. In terms of periodontal health, vitamin D_3_ impacts both the alveolar bone, which supports the tooth roots, and the periodontal tissues, including the gums. Sufficient levels of vitamin D_3_ promote the mineralization of the alveolar bone, preventing its resorption and reducing the risk of tooth loss associated with periodontal disease [4]. Furthermore, vitamin D3 may enhance the expression of key osteogenic markers such as runt-related transcription factor 2 (Runx2), alkaline phosphatase (ALP), bone gamma-carboxyglutamate protein (BGLAP), bone morphogenetic protein 2 (BMP2), and the vitamin D receptor (VDR) during the early stages of osteogenic differentiation in periodontal ligament stem cells (PDLSCs) [9].

Krall et al. [10] conducted a three-year, double-blind, randomized, placebo-controlled trial including 145 community-dwelling adults aged ≥65 years to evaluate the impact of supplementation on tooth retention. Participants were administered 700 IU of vitamin D_3_ and 500 mg of elemental calcium daily in a fixed oral regimen throughout the study period. Serum 25(OH)D concentrations were not monitored, and the authors did not report baseline or follow-up values; instead, they referred to their earlier work conducted by Dawson-Hughes et al. [12], in which identical doses produced a moderate increase in circulating 25(OH)D. After 3 years, the supplementation group exhibited an approximately 40% lower risk of tooth loss compared with placebo, independent of age, sex, baseline vitamin D status, or initial periodontal condition. These findings indicate that adequate vitamin D_3_ intake, in combination with calcium, may mitigate age-related alveolar bone resorption. However, this study was limited by the absence of serum 25(OH)D monitoring, relatively small sample size, and concomitant calcium supplementation, which precludes attribution of the observed effects solely to vitamin D_3_.

Importantly, the beneficial effects observed in periodontal tissues are consistent with evidence from large, randomized trials investigating skeletal bone metabolism. In the OSTPRE-FPS trial, Kärkkäinen et al. [13] conducted a three-year, population-based randomized study in women aged 65–71 years and demonstrated that combined calcium and vitamin D supplementation significantly attenuated bone mineral density (BMD) loss at clinically relevant skeletal sites, including the femoral neck and lumbar spine. The authors concluded that long-term supplementation contributed to the preservation of bone mass and reduction in age-related bone loss.

Given that alveolar bone shares similar remodeling dynamics and metabolic responsiveness to systemic bone as other skeletal sites, these findings support the biological plausibility that vitamin D_3_ and calcium supplementation may exert protective effects on periodontal bone. Together, data from both periodontal and osteoporotic research indicate that vitamin D_3_ plays a critical role in maintaining bone homeostasis, suggesting that systemic skeletal benefits may extend to the preservation of alveolar bone integrity in older adults.

## 3. Vitamin D_3_ and Immune Modulation

The biological effects of vitamin D_3_ in periodontal tissues are supported by varying levels of evidence, ranging from well-characterized molecular mechanisms to emerging and largely speculative pathways. The immune system plays a central role in the pathogenesis of periodontal disease, with both innate and adaptive immune responses contributing to tissue destruction (Figure 1). Calcitriol modulates immune function by influencing monocytes, macrophages, dendritic cells, and T lymphocytes [5,14].

Meghil et al. [15] explored this effect in a randomized controlled trial involving patients with moderate to severe periodontitis. Vitamin D_3_ supplementation (4000 IU/day for 16 weeks) led to increased serum 25(OH)D levels and decreased salivary concentrations of key pro-inflammatory cytokines, including interleukins (IL-1β, IL-6, IL-10) and tumor necrosis α (TNF-α). Furthermore, vitamin D downregulated CD3+, CD8+ T cell populations, suggesting an immunoregulatory effect. On a molecular level, it upregulated autophagy-related proteins (ATG5–12, ATG7, ATG16L1, Beclin-1), enhancing antimicrobial defenses.

Vitamin D_3_-mediated induction of antimicrobial peptides such as LL-37 (which enhance the epithelial barrier against microbial invasion) and modulation of pro-inflammatory cytokines (e.g., IL-1β, TNF-α) are well supported by experimental and clinical evidence [16,17]. The study by Figgins et al. [18] examined how vitamin D_3_ enhances innate immunity in gingival epithelial cells (GECs) by stimulating the expression of the antimicrobial peptide LL-37 and strengthening the epithelial barrier against microbial invasion (Table 1). This in vitro experimental study used human gingival epithelial cell lines (OKF6/TERT-1) and 3D primary gingival cultures (EpiGingiva). Cells were treated with vitamin D_3_, 25(OH)D_3_, or 1,25(OH)_2_D_3_ at concentrations around 100 nM (10^−7^ M) for 24 h. The researchers measured the expression of LL-37 and CYP24A1 genes by qRT-PCR, analysed prostaglandin E_2_ (PGE_2_) secretion as a marker of inflammation, and assessed bacterial invasion by Porphyromonas gingivalis and Filifactor alocis using confocal microscopy. The results showed that vitamin D_3_ alone increased LL-37 expression by 5–10-fold and significantly reduced PGE_2_ levels, indicating both antimicrobial and anti-inflammatory activity. Moreover, treatment with vitamin D_3_ markedly reduced bacterial invasion, suggesting that epithelial cells exposed to vitamin D develop stronger innate defences against oral pathogens. In summary, even inactive vitamin D_3_ can enhance gingival innate immunity by inducing LL-37 and reinforcing the epithelial barrier. The findings support the potential for developing topical vitamin D formulations as safe adjunctive therapies to prevent or control gingival inflammation and microbial invasion in the oral cavity.

Gingival epithelial cells exposed to vitamin D exhibit enhanced innate responses, potentially mitigating the pathogenic effects of oral biofilms [19,20].

## 4. Vitamin D_3_ and Gingivitis: Inflammatory and Clinical Associations

Gingivitis is the most prevalent and mild form of periodontal disease, characterized by gingival inflammation, bleeding on probing (BoP), and edema, primarily caused by the accumulation of microbial biofilm on tooth surfaces [21].

Although mechanical plaque control and professional prophylaxis remain the primary methods of prevention and treatment, recent evidence suggests that vitamin D_3_ supplementation may provide additional therapeutic benefits due to its immunomodulatory and anti-inflammatory properties. In a randomized, double-blind, placebo-controlled clinical trial, Srivastava et al. [22] demonstrated a clear dose-dependent effect of vitamin D_3_ on gingival inflammation: after 90 days of supplementation, the group receiving 2000 IU/day exhibited the greatest reduction in the gingival index (GI). Similar findings were reported by Hiremath et al. [23], who also observed that daily doses ranging from 500 to 2000 IU were safe and effective for reducing gingival inflammation, with higher doses producing more rapid improvements. Additional clinical studies and systematic reviews support these observations, reporting that vitamin D supplementation is associated with improved periodontal parameters and may enhance the outcomes of nonsurgical periodontal therapy. Mechanistically, these benefits are attributed to vitamin D_3_’s capacity to suppress pro-inflammatory cytokine production, modulate T-cell responses, and enhance innate immune defenses—effects that collectively contribute to the reduction in clinical signs of gingival inflammation [24,25].

The study conducted by Dietrich et al. [26], investigated the relationship between serum 25-hydroxyvitamin D [25(OH)D] levels and gingival inflammation in a large U.S. population (Table 2) It was a cross-sectional observational study, not randomized, based on data from NHANES III (1988–1994)—a nationally representative health and nutrition survey. The analysis included 6700 never-smokers aged 13–90 years, representing 77,503 gingival sites (teeth). To focus on gingivitis rather than periodontal disease, only teeth without attachment loss (≤2 mm) were analysed. Serum vitamin D levels were measured using a radioimmunoassay, and gingival inflammation was assessed by the presence or absence of BoP at each site. Results showed a clear inverse association between serum vitamin D and gingival bleeding. Compared with the lowest vitamin D quintile (median ≈ 32 nmol/L), participants in the highest quintile (median ≈ 100 nmol/L) were 20% less likely to bleed on probing (OR = 0.80; 95% CI 0.69–0.92; *p* < 0.001). The relationship was linear across the full range of vitamin D concentrations, with no threshold effect detected. The authors concluded that higher vitamin D status is associated with lower susceptibility to gingival inflammation, likely through anti-inflammatory and immunomodulatory effects, showing that the active form of vitamin D-1,25(OH)_2_D_3_, inhibits T-cell proliferation and the production of pro-inflammatory cytokines (IL-2, IFN-γ), and also affects the maturation of dendritic cells, which explains its potential immunoregulatory role in gingival tissues.

A prospective interventional study conducted by Gurbanov et al. [27] included 101 systemically healthy adults diagnosed with vitamin D deficiency (<20 ng/mL) evaluated whether restoring serum vitamin D levels could influence matrix metalloproteinase-9 (MMP-9) levels in the gingival crevicular fluid (GCF)—a biochemical marker linked to gingivitis. Participants were divided into three equal groups based on periodontal status: 33 with periodontal health, 34 with gingivitis, and 34 with periodontitis. All were nonsmokers, had not undergone periodontal treatment in the previous six months, and were free from systemic medications or conditions that might affect periodontal status.

Furthermore, vitamin D deficiency is associated with an increased susceptibility to periodontal infections, partly due to impaired production of the antimicrobial peptide LL-37. Figgins et al. [18] demonstrated that inactive vitamin D_3_, when combined with histone deacetylase (HDAC) inhibitors such as sodium butyrate, phenylbutyrate, or MS-275, significantly upregulated LL-37 expression in oral keratinocytes and three-dimensional gingival epithelial models. This combination not only amplified antimicrobial defense but also reduced PGE2 levels, attenuating local inflammation. The synergistic use of vitamin D_3_ and HDAC inhibitors was particularly effective in reducing invasion by Porphyromonas gingivalis and Filifactor alocis, indicating promising adjunctive strategies for managing gingivitis.

## 5. Vitamin D_3_ and Periodontitis: Inflammatory and Clinical Associations

Periodontitis is a chronic inflammatory condition leading to progressive destruction of the supporting structures of the teeth. It has been closely linked to vitamin D_3_ deficiency, which may exacerbate disease progression and compromise the effectiveness of treatment.

A monocentric, cross-sectional study conducted at the Jagiellonian University in Cracow [28] evaluated serum 25(OH)D_3_ levels in 100 adults with and without periodontitis. All participants were systemically healthy and had similar body mass indices. Periodontal status was assessed using clinical attachment level (CAL), probing pocket depth (PPD), BoP, and plaque index (PI). Individuals diagnosed with periodontitis exhibited significantly lower serum vitamin D_3_ levels compared to healthy controls. Moreover, an inverse correlation was observed between serum 25(OH)D_3_ concentration and the severity (stage I–IV) and extent (localized vs. generalized) of periodontitis. Vitamin D levels below 75 nmol/L were associated with worse clinical attachment loss.

### 5.1. Vitamin D_3_ Supplementation and Nonsurgical Periodontal Therapy Outcomes

This subsection integrates the anti-inflammatory and immunomodulatory mechanisms discussed above with clinical evidence evaluating the impact of vitamin D_3_ supplementation on nonsurgical periodontal therapy outcomes.

Vitamin D_3_ supplementation may enhance the outcomes of nonsurgical periodontal therapy by reducing PPD and promoting healing. In a randomized, double blind, placebo controlled trial by Perić et al. [29], 27 patients with periodontitis and vitamin D deficiency (baseline serum 25 OH D_3_ below 30 ng/mL) were randomized to receive either weekly supplementation of 25,000 IU of vitamin D_3_ or placebo for six months, beginning one month prior to SRP. In this study, both groups showed clinical improvement, but the vitamin D-supplemented group demonstrated a greater reduction in PPD. Moreover, BoP-measured as full mouth bleeding score (FMBS)—also decreased in both groups, but the test group tended towards a lower bleeding score, indicating a stronger anti-inflammatory effect. Limitations include the small sample size and restriction to vitamin D–deficient patients, limiting generalizability to individuals with sufficient baseline vitamin D levels.

Vitamin D_3_ plays a significant role in supporting periodontal regeneration, partly through the upregulation of BMP-2, a key factor in osteogenesis. A randomized, 6-month clinical trial by Lei et al. [30] investigated the efficacy of vitamin D_3_ supplementation as an adjunct to non-surgical periodontal therapy in 60 adults with periodontitis, type 2 diabetes, and vitamin D deficiency (25(OH)D_3_ < 30 ng/mL). Participants received standard periodontal treatment (scaling, root planing, oral hygiene instruction) and were assigned to either a low-dose (25,000 IU/week) or high-dose (50,000 IU/week) vitamin D_3_ group. Both groups demonstrated clinical improvement; however, the high-dose group showed significantly better outcomes. The high-dose group demonstrated consistently greater improvements in PPD, CAL, bleeding index, and plaque index compared to the low-dose group. Notably, BMP-2 levels in gingival crevicular fluid increased in a dose-dependent manner: from ~30 pg/mL at baseline to 167.03 pg/mL (low-dose) and 499.51 pg/mL (high-dose) after 6 months. These findings suggest that high-dose vitamin D_3_ supplementation significantly enhances clinical outcomes of non-surgical periodontal therapy in vitamin D–insufficient patients, potentially via BMP-2–mediated periodontal regeneration. Vitamin D_3_ may serve as a valuable adjunct in managing periodontitis, particularly in systemically compromised populations. The study population was limited to patients with type 2 diabetes and vitamin D deficiency, and the follow-up period was relatively short, which should be considered when interpreting the clinical relevance of the findings.

### 5.2. Vitamin D_3_ Status and Surgical Periodontal Therapy Outcomes

This subsection further extends the clinical relevance of vitamin D_3_ by examining how baseline vitamin D status influences healing, inflammation resolution, and bone regeneration following surgical periodontal therapy.

The influence of baseline vitamin D_3_ levels on the effectiveness of surgical periodontal procedures has also been explored. Bashutski et al. [31] conducted a prospective study on 40 patients undergoing open flap debridement. All participants received 800 IU of vitamin D_3_ and 1000 mg of calcium daily starting three days prior to surgery and continuing for six weeks postoperatively. Over a 12-month follow-up, CAL, PD, and bone defect healing were assessed. Patients with sufficient vitamin D_3_ levels showed significantly greater improvements in CAL and PD compared to those with deficient levels (CAL gain: 0.92 mm vs. −0.43 mm; PD reduction: 1.83 mm vs. 0.43 mm, *p* < 0.01). These findings underscore the importance of optimal vitamin D status for favorable outcomes following surgical periodontal interventions.

For instance, a prospective observational study [31] demonstrated that patients with sufficient serum 25 OH D experienced significantly greater probing depth reduction and clinical attachment gain at 6 and 12 months post flap surgery compared to vitamin D–deficient patients. Moreover, cross sectional epidemiological data (e.g., NHANES) indicate a strong negative association between vitamin D_3_ levels and periodontal attachment loss, underscoring the potential importance of vitamin D in periodontal tissue maintenance and healing. These findings reinforce the notion that optimizing vitamin D status may be beneficial in the context of surgical periodontal therapy [32,33].

## 6. Periodontal Pocket Depth and Nonsurgical Treatment Outcomes

In recent years, there has been a noticeable increase in studies investigating the relationship between vitamin D_3_ levels and periodontal health. Due to the immunomodulatory and anti-inflammatory properties of this vitamin, many researchers have begun to explore its potential impact on the progression of periodontal diseases and the effectiveness of periodontal therapy. Vitamin D_3_ is also known for its immunomodulatory properties. Its active form, 1,25-dihydroxyvitamin D_3_, exerts its effects through the VDR, which is expressed in most cells of the body, including periodontal cells. Human Periodontal ligament stem cells (hPDLSCs), osteoblasts, and macrophages respond to vitamin D_3_ under both physiological and inflammatory conditions [34].

In a study conducted by Blufstein et al. [35] there were examined the effects of two forms of vitamin D_3_-1,25-dihydroxyvitamin D_3_ and 25-hydroxyvitamin D_3_, on the osteogenic differentiation of hPDLSCs, and its therapeutic alteration under inflammatory conditions simulated by *P. gingivalis* LPS or Pam3Cys-Ser-(Lys)-4 (Pam3CSK4). The experiment aimed to determine how inflammation affects vitamin D_3_-induced osteogenic differentiation of hPDLSCs. The laboratory-based in vitro experimental study involving primary cell cultures derived from human donors including 5 individuals within 18–22 age, periodontally healthy; without chronic illnesses or regular medications, whose third lower molars were extracted due to orthodontic reasons. Examined tissue was harvested from the middle third of extracted tooth roots and searched for hPDLSCs, characterized by flow cytometry as mesenchymal stromal cells (positive for CD29, CD90, CD105, CD146; negative for CD14, CD31, CD34, CD45). Cells were cultured in osteogenic induction medium containing dexamethasone, β-glycerophosphate, and ascorbic acid. They were stimulated with either 1,25-dihydroxyvitamin D_3_ (0, 0.1, 10 nM) or 25-hydroxyvitamin D_3_ (0, 1, 100 nM), with or without inflammatory stimuli: *P. gingivalis* LPS or Pam3CSK4, for 7, 14, and 21 days. Treatment with 1,25-dihydroxyvitamin D_3_ (10 nM) significantly promoted osteogenic differentiation of hPDLSCs, increasing mineral deposition (*p* < 0.01) and upregulating osteocalcin and osteopontin expression (*p* < 0.05). Inflammatory stimuli, especially Pam3CSK4, partially suppressed this gene expression, though mineralization remained unaffected. Runx2 expression did not change significantly under any condition. In contrast, 25-hydroxyvitamin D_3_ had no significant effect on osteogenesis. These results suggest that while active vitamin D_3_ supports osteogenic activity in periodontal ligament cells, inflammatory conditions can reduce its gene-level effects without impairing mineral formation.

Another study performed by Liu et al. [36] aimed to evaluate the expression of CYP27B1 (vitamin D 1α-hydroxylase) in gingival fibroblasts in vivo, and to determine whether this expression is associated with periodontal inflammation in patients with Stage IV Grade C periodontitis, compared to healthy controls. 75 patients (42 in periodontal group- mean 33.5 ± 7.8 years and 33 healthy individuals undergoing crown-lengthening surgery- mean 30.0 ± 9.2 years) took part in that trial. Participants were non-smokers without any systemic diseases or pregnancy. Gained samples were divided into immunohistochemistry and immunofluorescence (protein detection) and real-time PCR (mRNA expression analysis). Expression of CYP27B1 was assessed in both gingival epithelia and connective tissues. Co-localization with vimentin confirmed that CYP27B1 was expressed in fibroblasts. Staining intensity was scored from 0 to 3 by two blinded pathologists. The study demonstrated that CYP27B1, the key enzyme responsible for converting 25-hydroxyvitamin D_3_ into its active form, is expressed in both the epithelial and connective tissues of human gingiva, with significantly higher expression levels observed in patients with Stage IV Grade C periodontitis compared to healthy controls. Quantitative PCR revealed elevated CYP27B1 mRNA levels in the gingival connective tissues of periodontitis patients, while no significant differences were found in epithelial tissues. Immunohistochemical analysis confirmed strong protein expression of CYP27B1 in fibroblasts, and immunofluorescence staining showed its colocalization with vimentin, verifying that gingival fibroblasts were the primary source of this enzyme. Nearly all fibroblasts in the diseased samples were CYP27B1-positive, and the staining score was significantly higher in the periodontitis group (2.49 ± 0.08) compared to controls (1.84 ± 0.12). These findings confirm the in vivo expression of CYP27B1 in human gingival fibroblasts and suggest that its expression is positively associated with periodontal inflammation. This supports the hypothesis that the vitamin D pathway plays a role in the local immune defense of periodontal tissues and may contribute to the pathophysiology of periodontitis. Thus, activation of this pathway-potentially through vitamin D supplementation, could represent a therapeutic avenue in managing periodontal disease.

The examination conducted by Foey et al. [14] aimed to determine whether *P. gingivalis* differentially induces endotoxin tolerance in these macrophage subsets. To do this, THP-1 monocytes were differentiated into M1-like macrophages using PMA and into M2-like macrophages using 1,25-dihydroxyvitamin D3. Vitamin D3 was used specifically to promote an immunoregulatory M2 phenotype, mimicking the homeostatic conditions of the oral mucosa. The study evaluated whether M2 macrophages induced by vitamin D3 are more susceptible to *P. gingivalis*-mediated suppression of inflammatory responses compared to pro-inflammatory M1 macrophages. The macrophages were pre-treated with either *P. gingivalis* LPS, heat-killed PG (HKPG), or other bacterial ligands such as LTA and PGN, followed by stimulation to assess cytokine responses and nuclear factor kappa-light-chain-enhancer of activated B cells (NFκB) activation. Cytokines measured included TNFα, IL-1β, IL-6, and IL-10, using ELISA, while NFκB activity was assessed via a reported assay. The results showed that M1 and M2 macrophages responded differently to *P. gingivalis* stimulation. M1 cells exhibited a strong pro-inflammatory cytokine profile (TNFα:IL-1β:IL-6 ratio of 99:2:1), while M2 cells had a more balanced, lower-level response (ratio of 4:2:1). Importantly, *P. gingivalis* induced significant endotoxin tolerance in vitamin D3-induced M2 macrophages—reducing TNFα by up to 94% (*p* = 0.0383)—as well as in CD14^lo M1 cells. In contrast, CD14^hi M1 macrophages, which represent inflammatory monocyte-derived cells recruited during disease, were resistant to this suppression. NFκB activation patterns mirrored cytokine profiles, further supporting functional differences between the subsets. IL-10, a key anti-inflammatory cytokine, was predominantly produced by CD14^lo M2 cells (up to 124 pg/mL), and minimally by CD14^hi M1 cells (~41–43 pg/mL). *P. gingivalis* LPS also demonstrated lower endotoxin activity compared to *E. coli* LPS, especially in M1 cells. *P. gingivalis* selectively induces endotoxin tolerance in vitamin D3-induced M2 macrophages and CD14^lo M1 cells, while sparing CD14^hi M1 pro-inflammatory macrophages. This selective suppression may allow the pathogen to evade immune clearance while sustaining chronic inflammation and tissue destruction characteristics of periodontitis. The study highlights the importance of macrophage subset-specific responses—and the role of vitamin D3 in shaping them—in understanding host–pathogen interactions and developing targeted therapies for chronic inflammatory diseases.

The active form of vitamin D, known as 1,25-dihydroxyvitamin D_3_, can induce the production of antimicrobial peptides and reduce the expression of pro-inflammatory cytokines, offering both protective and healing properties within oral tissues. Emerging evidence suggests that gingival epithelial cells may have the capacity to locally activate vitamin D, opening the door for topical vitamin D therapies as a novel approach in periodontal disease prevention and treatment. Menzel et al. [17] investigated in their trial vitamin D’s influence on oral immune defense and tissue protection, using both mouse models and human GEC. Mice rendered vitamin D deficient through a specialized diet developed clear signs of gingival inflammation and alveolar bone loss, mimicking human periodontitis. In vitro experiments demonstrated that treating GECs with active vitamin D reduced the intracellular presence of *P. gingivalis*, a key bacterial pathogen in periodontitis, confirming an enhanced innate immune response. In this study, wild-type C57Bl/6 mice were placed on a vitamin D-deficient diet for six weeks. Compared to controls, these mice had a significant drop in serum 25(OH)D_3_ levels, developed pronounced gingival inflammation, and exhibited measurable alveolar bone loss. MicroCT imaging revealed a significant reduction in bone volume in vitamin D-deficient mice (*p* < 0.01), and histological analysis showed a marked increase in TRAP-positive osteoclasts (*p* < 0.001), confirming active bone resorption. The gingiva also showed significantly higher inflammation scores, indicating the onset of periodontal disease due to vitamin D deficiency. In vitro, both primary GECs and immortalized OKF6/TERT1 cells treated with 10 nM 1,25(OH)_2_D_3_ showed a statistically significant reduction in intracellular Porphyromonas gingivalis. In primary GECs, fluorescence intensity (measuring intracellular bacteria) decreased by over 50% compared to control (*p* < 0.05). A similar decrease was observed in OKF6/TERT1 cells. This confirmed that active vitamin D enhances antibacterial defense in gingival cells. Additionally, gingival cells were shown to express key enzymes needed for vitamin D activation: CYP2R1, CYP27A1, and CYP27B1. Enzyme assays confirmed the conversion of vitamin D_3_ to 25(OH)D_3_ within 2 h, and of 25(OH)D_3_ to 1,25(OH)_2_D_3_ within 6 h after exposure. This confirmed that gingival epithelial cells can indeed locally activate inactive vitamin D. Vitamin D treatment also significantly reduced inflammation-related gene expression. In vitro treatment of oral epithelial cells with 10 nM 1,25(OH)_2_D_3_ led to a >2-fold reduction in IL-1α mRNA expression. In vivo, mice treated topically with 50 μL of 1 μM vitamin D_3_ or 1,25(OH)_2_D_3_ showed a rapid and significant decrease in IL-1α mRNA in the gingiva within 6 h of application (*p* < 0.0001), demonstrating the anti-inflammatory effect of both inactive and active vitamin D applied locally.

Importantly, heterogeneity in the clinical literature reflects real-world differences in patient populations rather than solely methodological limitations. Studies vary substantially with respect to baseline vitamin D status, systemic health, age, supplementation dose, co-administration of calcium, and duration of follow-up. Many interventional trials focus on vitamin D-deficient or systemically compromised individuals, which may partially explain the variability in reported outcomes. Therefore, the clinical effects of vitamin D_3_ supplementation should not be assumed to be uniform across all periodontal patients.

## 7. Vitamin D_3_ in Surgical Periodontal Therapy

The anti-inflammatory effects of vitamin D_3_ are helpful in periodontal treatment, influencing the immune response, and enhancing tissue healing. Its deficiency is associated with worse periodontal outcomes such as increased pocket depth and attachment loss. The study conducted by M and Aboelsaad [37] aimed to prove properties and indicate a proper dose of D_3_ intake succeeding in improvement of periodontal health (Table 3). The study involved 28 patients aged between 30 and 45 years (14 men and 14 women), all of whom were smokers and had mild to moderate chronic periodontitis with serum vitamin D levels below 30 ng/mL. Participants were randomly assigned to one of two groups: a test group that received 10,000 IU of oral vitamin D_3_ daily (five days a week) for 12 weeks along with SRP, and a control group that received placebo pills with the same SRP procedure. Both patients and clinicians were blinded to the group assignments. The study lasted for 12 weeks. Clinical periodontal parameters—GI, PI, PPD, and CAL—were measured at baseline and after three months. The results showed that both groups experienced improvements, but the test group had significantly greater reductions in all periodontal parameters. Specifically, the test group showed a 33.35% reduction in GI, 29.43% in PI, 45.00% in PPD, and 32.71% in CAL. In contrast, the control group showed smaller improvements: 14.7% in GI, 19.5% in PI, 18.9% in PPD, and 19.5% in CAL. The most significant difference between groups was in PPD, where the test group achieved a 26.07% greater reduction than the control group (*p* < 0.05). These dosing regimens were evaluated within specific clinical contexts and should not be interpreted as universal treatment recommendations.

It should be emphasized that high-dose vitamin D_3_ supplementation should be undertaken with caution. The tolerable upper intake level for adults is generally set at 4000 IU/day, although higher doses may be used short term under medical supervision. Excessive vitamin D intake may lead to hypercalcemia or vascular calcification, particularly in patients with renal disease, granulomatous disorders, or hyperparathyroidism. Therefore, supplementation strategies should be individualized and guided by serum 25(OH)D monitoring.

## 8. Summary

Vitamin D_3_, a fat-soluble steroid, plays a pivotal role in maintaining periodontal health through its effects on bone metabolism, immune modulation, and inflammation control. The active form, 1,25-dihydroxyvitamin D_3_, interacts with the VDR expressed in periodontal cells, including hPDLSCs, osteoblasts, fibroblasts, and macrophages, influencing both physiological and inflammatory processes. Adequate vitamin D_3_ levels promote alveolar bone mineralization, upregulate osteogenic markers such as Runx2, ALP, BMP2, and enhance periodontal regeneration.

Furthermore, vitamin D_3_ modulates the innate and adaptive immune responses by upregulating antimicrobial peptides like LL-37, downregulating pro-inflammatory cytokines (e.g., IL 1β, TNF α, IL 6), and promoting anti-inflammatory T-cell responses, contributing to reduced gingival inflammation and improved healing outcomes. Clinical and experimental evidence demonstrates that both systemic supplementation and local activation of vitamin D_3_ can enhance periodontal therapy outcomes, including reductions in PPD, CAL, and BoP. Table 4 summarizes the main mechanisms by which vitamin D_3_ exerts these effects in periodontal disease [16,20,38,39,40,41,42,43,44].

Dose-dependent studies indicate that higher doses of vitamin D_3_ supplementation (e.g., 10,000 IU/day or 50,000 IU/week) result in significantly greater improvements in periodontal parameters compared to standard doses, highlighting the importance of correcting deficiencies for therapeutic efficacy. Evidence also supports a role for vitamin D_3_ in surgical periodontal interventions, where sufficient baseline vitamin D levels correlate with better clinical attachment gain, pocket depth reduction, and bone healing.

Available evidence suggests that the benefits of vitamin D_3_ supplementation are most consistent in individuals with suboptimal or deficient serum 25(OH)D levels. In populations with sufficient baseline vitamin D status, the additional periodontal benefits of supplementation appear less pronounced and remain inconsistent across studies. Importantly, many clinical trials did not stratify outcomes according to baseline vitamin D levels, limiting conclusions regarding supplementation effects in vitamin D–replete individuals. This represents an important gap in the current literature.

## 9. Limitations

This review has several limitations—as a narrative review, it does not follow a systematic methodology and therefore may be subject to selection bias. Although a structured literature search was performed, the inclusion of studies was not based on predefined eligibility criteria or formal risk-of-bias assessment. Also the available evidence is heterogeneous, encompassing in vitro experiments, animal models, observational studies, and interventional clinical trials with varying designs, supplementation protocols, and outcome measures. This heterogeneity limits direct comparison between studies and precludes quantitative synthesis. Finally, while associations between vitamin D_3_ status and periodontal health are well supported, causal relationships and standardized therapeutic recommendations require further validation through large-scale, well-designed randomized controlled trials.

## 10. Conclusions

Vitamin D_3_ is a multifaceted adjunct in the prevention and management of periodontal diseases. Its combined effects on bone metabolism, immune regulation, and anti-inflammatory pathways position it as a key modulator of periodontal health. Both experimental and clinical data indicate that adequate systemic levels, and potentially local activation of vitamin D_3_ in gingival tissues, can enhance outcomes of nonsurgical and surgical periodontal therapies. Correcting vitamin D deficiency should therefore be considered an integral component of periodontal disease management.

Future research should focus on optimizing dosing strategies, exploring topical applications, and further elucidating molecular mechanisms (as outlined in Table 4) to fully harness the therapeutic potential of vitamin D_3_ in periodontal care.

## Figures and Tables

**Table 1:** Immunomodulatory effects of Vitamin D in oral cavities.

Dosage of Administered Vitamin D3, Its Form and Adjuvants	Effect After Absorption	Type of Molecules	References
100 µg Vitamin D_3_/day for 16 weeks	Decreased salivary concentrations of key pro-inflammatory cytokines	IL-1β, TNF-α, IL-6, IL-10	[15]
100 µg Vitamin D_3_/day for 16 weeks	Downregulation of lymphocytes suggesting an immunomodulatory effect	T-cells: CD3+, CD8+	[15]
100 µg Vitamin D_3_/day for 16 weeks	Upregulation of autophagy-related proteins and therefore enhance antimicrobial defenses	ATG5–12, ATG7, ATG16L1, Beclin-1	[15]
Vitamin D_3_ (cholecalciferol), 25(OH)D_3_, or 1,25(OH)_2_D_3_ at concentrations around 100 nM (10^−7^ M) for 24 h	Increased antimicrobial peptide expression resulting in antimicrobial activity	LL-37 and CYP24A1	[19,20]
Vitamin D_3_ (cholecalciferol), 25(OH)D_3_, or 1,25(OH)_2_D_3_ at concentrations around 100 nM (10^−7^ M) for 24 h	Significantly reduced prostaglandin levels resulting in anti-inflammatory activity	PGE2	[19,20]
Vitamin D_3_ (cholecalciferol), 25(OH)D_3_, or 1,25(OH)_2_D_3_ at concentrations around 100 nM (10^−7^ M) for 24 h	Reduced bacterial invasion by reinforcing epithelial barrier	Epithelial cells	[19,20]

**Table 2:** Clinical effects of vitamin D_3_ supplementation on gingivitis parameters.

Gingivitis Parameter	Effect of Vitamin D_3_	Vitamin D_3_ Doses Associated with Improvement	References
Gingival Index (GI)	Reduction in inflammation; clear dose–response effect	25–50 µg/day; strongest effect at 50 µg/day	[22,23]
Bleeding on probing (BoP)	Lower bleeding susceptibility	Higher serum 25(OH)D → 20% lower BoP risk	[26]
Gingival erythema and edema	Noticeable reduction in inflammatory signs	All doses: 25–50 µg/day	[22,23]

**Table 3:** Dose-dependent reduction in periodontal parameters while Vitamin D3 intake [37].

Clinical Periodontal Parameters Reduction	Test Group That Received 10,000 IU of Oral Vitamin D_3_ Daily for 12 Weeks	Control Group That Received Placebo Pills Daily for 12 Weeks	Reduction Difference
GI	33%	15%	18%
PI	29%	20%	9%
PPD	45%	19%	26%
CAL	33%	20%	13%

GI—gingival index; PI—plaque index; PPD—probing pocket depth; CAL—clinical attachment loss.

**Table 4:** Mechanisms of Action of Vitamin D3 in Periodontal Disease.

Mechanism	Description	References
Modulation of microbial biofilm	Alters the composition of the oral microbiome, reducing pathogenic species associated with periodontitis	[38,39,40,41]
Immunomodulation	Modulation of innate and adaptive immune responses, including suppression of pro-inflammatory cytokines and promotion of IL-10	[20,40,42]
Regulation of T-cell function	Promotes regulatory T cell development and inhibits Th17 responses, which are implicated in periodontal inflammation	[20,40]
Promotion of epithelial barrier integrity	Strengthens epithelial cell junctions, improving mucosal defense against bacterial invasion	[20,40]
Inhibition of osteoclastogenesis	Reduces RANKL expression and increases osteoprotegrin (OPG) levels, inhibiting bone resorption	[42,43]
Reduction in oxidative stress	Enhance antioxidant enzyme activity and reduces oxidative damage in periodontal tissue	[16,43]
Reduction in systemic and local inflammation	Decreases levels of CRP and pro-inflammatory markers in both serum and gingival crevicular fluid	[16,43,44]

## Data Availability

No new data were created or analysed in this study. Data sharing is not applicable to this article.

## Abbreviations

The following abbreviations are used in this manuscript:

VDR	Vitamin D Receptor
PPD	Probing Pocket Depth
CAL	Clinical Attachment Loss
7-DHC	7-Dehydrocholesterol
Runx2	Runt-related Transcription Factor 2
ALP	Alkaline Phosphatase
BGLAP	Bone Gamma-carboxyglutamate Protein
BMP2	Bone Morphogenetic Protein 2
PDLSCs	Periodontal Ligament Stem Cells
BMD	Bone Mineral Density
TNF-α	Tumor Necrosis α
LL-37	Cathelicidin
GECs	Gingival Epithelial Cells
PGE_2_	Prostaglandin E_2_
BoP	Bleeding on Probing
GI	Gingival Index
MMP-9	Matrix Metalloproteinase-9
GCF	Gingival Crevicular Fluid
HDAC	Histone Deacetylase
PI	Plaque Index
FMBS	Full-mouth Bleeding Score
BI	Bleeding Index
hPDLSCs	Human Periodontal Ligament Stem Cells
Pam3CSK4	Pam3Cys-Ser-(Lys)-4
HKPG	Heat-killed PG
NFκB	Nuclear Factor Kappa-light-chain-enhancer of Activated B Cells
OPG	Osteoprotegrin
